# A novel posterior occlusal splint improves symptoms and prognosis of coexisting temporomandibular disorders and deep bite: a retrospective study

**DOI:** 10.1007/s00784-026-06772-4

**Published:** 2026-02-09

**Authors:** Yunyi Yuan, Antong Wu, Jiaqian Fan, Yufu Lin, Zhilei Liu, Liwen Huang, Junxi Feng, Qingbin Zhang, Zhenlong Liu, Xingyang Li, Wei Cao

**Affiliations:** 1 Department of Temporomandibular Joint, School and Hospital of Stomatology, Guangdong Engineering Research Center of Oral Restoration and Reconstruction, Guangzhou Key Laboratory of Basic and Applied Research of Oral Regenerative Medicine, Guangzhou Medical University, Dongfengxi road 195, Guangzhou, 510180 China; 2 Orthodontics and Pediatric Dentistry Center, School and Hospital of Stomatology, Guangdong Engineering Research Center of Oral Restoration and Reconstruction, Guangzhou Key Laboratory of Basic and Applied Research of Oral Regenerative Medicine, Guangzhou Medical University, Guangzhou, China; 3 Laboratory for Myology, Department of Human Movement Sciences, Faculty of Behavioural and Movement Sciences, Vrije Universiteit Amsterdam, Amsterdam Movement Science, Amsterdam, The Netherlands; 4 Division of Dentistry, School of Medical Sciences, Faculty of Biology, Medicine and Health, The University of Manchester, Manchester, UK; 5 Henry Royce Institute, The University of Manchester, Manchester, UK

**Keywords:** Overbite, Temporomandibular joint disorders, Occlusal splints, Face pain, Temporomandibular joint dysfunction syndrome, Cone-Beam computed tomography

## Abstract

**Objectives:**

Temporomandibular disorders (TMD) are a common syndrome characterized by pain and mandibular dysfunction, with occlusal factors such as deep bite potentially contributing to biomechanical changes in the temporomandibular joints (TMJ). Managing patients with coexisting TMD and deep bite is often complex. This study aimed primarily to evaluate the clinical effectiveness of a novel posterior occlusal splint in improving TMD-related pain and mandibular function, and secondarily to assess overall treatment prognosis and changes in overbite.

**Materials and methods:**

This retrospective study included 448 patients (355 females, 93 males) with TMD and deep bite, aged 11–66 years (mean ± SD: 26.53 ± 9.93), all with full permanent dentition from the first premolar to the first molar. All patients were treated using a novel posterior occlusal splint. Clinical parameters-including joint tenderness, joint movement pain, push-back mandibular test results, maximum mouth opening, joint noises, and intermittent closed lock-were assessed at baseline (T0) and 1-, 3-, and 6-month follow-ups. Overbite measurements were taken using cone-beam computed tomography (CBCT) at T0) and at the 6-month follow-up (T6).

**Results:**

The splint significantly alleviated TMD-related pain, including joint tenderness, joint movement pain, and positive push-back mandibular test results, and demonstrated a stable long-term prognosis with low recurrence rates (2.6%–5%). Improvements were also observed in maximum mouth opening. No significant changes were found in joint noises, intermittent closed lock, or overbite values, with joint noises showing occasional recurrence (7%–10%).

**Conclusion:**

The novel posterior occlusal splint demonstrated promising short-term and sustained benefits in treating pain and improving mandibular function in patients with coexisting TMD and deep bite. However, its effectiveness in treating joint noises and deep bite appears limited.

**Clinical relevance:**

This splint offers a clinically effective, patient-friendly alternative to traditional occlusal appliances, particularly during the early stages of treatment for patients coexisting with TMD and deep bite.

## Introduction

Temporomandibular disorder (TMD) is a significant public health concern, affecting up to 7% of adolescents and 16% of adults [1, 2]. It encompasses a range of conditions characterized by pain and dysfunction of the masticatory system, including the temporomandibular joints (TMJ) and associated muscles [3]. Common clinical manifestations include muscle tenderness, restricted mandibular movement, and joint noises. Among these, pain and dysfunction are particularly disruptive, often interfering with daily activities and reducing the quality of life [4].

The etiology of pain-related TMD remains under debate, particularly regarding the role of occlusal factors as potential causative agents [5–12]. Although no definitive consensus exists, most TMD patients have varying degrees of malocclusion. Among these malocclusions, deep bite has received significant attention due to its potential biomechanical impact on the TMJ. Deep bite is defined as an excessive vertical overlap of the incisors, where the upper incisors cover more than one-third of the clinical crown of lower incisors in centric occlusion [13]. Studies have reported a high prevalence of deep bite among TMD patients, ranging from 18% to 54% [14, 15]. Khayat et al. [14] found that 23% of TMD patients exhibited severe deep bite, while over 70% were mild to moderate cases, while Williamson et al. [15] reported a 54.2% prevalence. These findings highlight a notable coexistence, indicating a potential correlation between the two conditions [6, 16].

Previous studies have suggested that deep bite may contribute to condylar overclosure, thereby increasing mechanical loading on the masseter and posterior temporalis muscles, triggering spasms in the lateral pterygoid muscles, potentially leading to anterior disc displacement [17]. Demir et al. [18] observed that short-term acupuncture significantly increased first molar bite force in patients with deep bite, suggesting the involvement of muscle dysfunction in TMD-related biomechanical alterations. Furthermore, studies have shown that deep bite is associated with mandibular stiffness, muscle disorders, and elevated somatization scores, all of which are linked to orofacial pain [19]. Consequently, TMD patients with coexisting deep bite are more likely to present with pain-related joint symptoms, possibly due to increased biomechanical stress on the TMJ and masticatory muscles, thereby contributing to the development or exacerbation of pain-related TMD.

Given this potential relationship, investigating interventions that alleviate TMD symptoms and assess changes in deep bite is clinically important.

Management of patients presenting with both TMD and deep bite is multifaceted, often requiring a multidisciplinary approach. The primary goals are to optimize orofacial muscle function, promote symmetrical and controlled mandibular movements, enhance TMJ lubrication, reduce hyperactivity in the elevator muscles, reestablish proper mandibular posture, and relieve pain [20]. As pain and restricted mouth opening are the most common complaints, effective strategies targeting these symptoms are essential.

Occlusal splint therapy remains the most commonly employed conservative approach and includes various splint designs. Splints are generally classified into three main types: relaxation/stabilization splints, distraction/pivot splints, and repositioning splints [21, 22]. Among these, stabilization splints are most widely adopted. While effectively reducing TMJ pain and improving mouth opening, full-arch stabilization occlusal splints often cause a pronounced foreign body sensation during wearing [23–25]. The widely used stabilization splints, due to their relatively large size, often cause patient discomfort and limited long-term compliance. To address these limitations, our research team developed a novel posterior occlusal splint based on the design principles of stabilization splints, incorporating key modifications to enhance comfort and wearability. By reducing the overall size of the appliance, this splint provides greater ease of use while maintaining therapeutic effectiveness. Functionally, it separates the upper and lower posterior dentition to eliminate abnormal occlusal interferences, thereby reducing intra-articular pressure and alleviating stress on the masticatory muscles. In our prior study, this novel splint demonstrated comparable TMD-related pain relief to stabilization splints and superior efficacy in increasing maximum mouth opening (MMO), particularly in early treatment stages [26]. However, that study did not focus on malocclusion types or longitudinal symptom progression. Moreover, in previous studies, post-treatment changes in occlusal characteristics have predominantly been assessed via two-dimensional or subjective methods, constraining measurement accuracy. Consequently, the full scope of occlusal splint effects on both TMD symptoms and coexisting malocclusion remains inadequately defined.

Based on these considerations, this study retrospectively reviewed patients with coexisting TMD and deep bite treated with this splint, using CBCT to quantify changes in deep bite and allow precise three-dimensional assessment. We hypothesized that the novel posterior occlusal splint could alleviate TMD-related symptoms, particularly joint pain, improve maximum mouth opening, and potentially affect the characteristics of deep bite and symptom progression. To test this hypothesis, we aimed to: (1) evaluate its effectiveness in alleviating TMD-related symptoms, particularly joint pain and limited MMO; (2) analyze symptom progression and prognosis throughout the treatment process, (3) and assess changes in anterior overbite using CBCT measurements. Understanding the impact of this splint on both TMD symptoms and deep bite has important clinical implications, potentially guiding conservative management strategies and informing personalized treatment planning for patients with these coexisting conditions.

## Materials and methods

### Participants

This retrospective cohort study reviewed the medical records of 448 patients who visited the Department of Temporomandibular Joint at the Affiliated Stomatology Hospital of Guangzhou Medical University between January 2019 and December 2024.

**Fig. 1:**
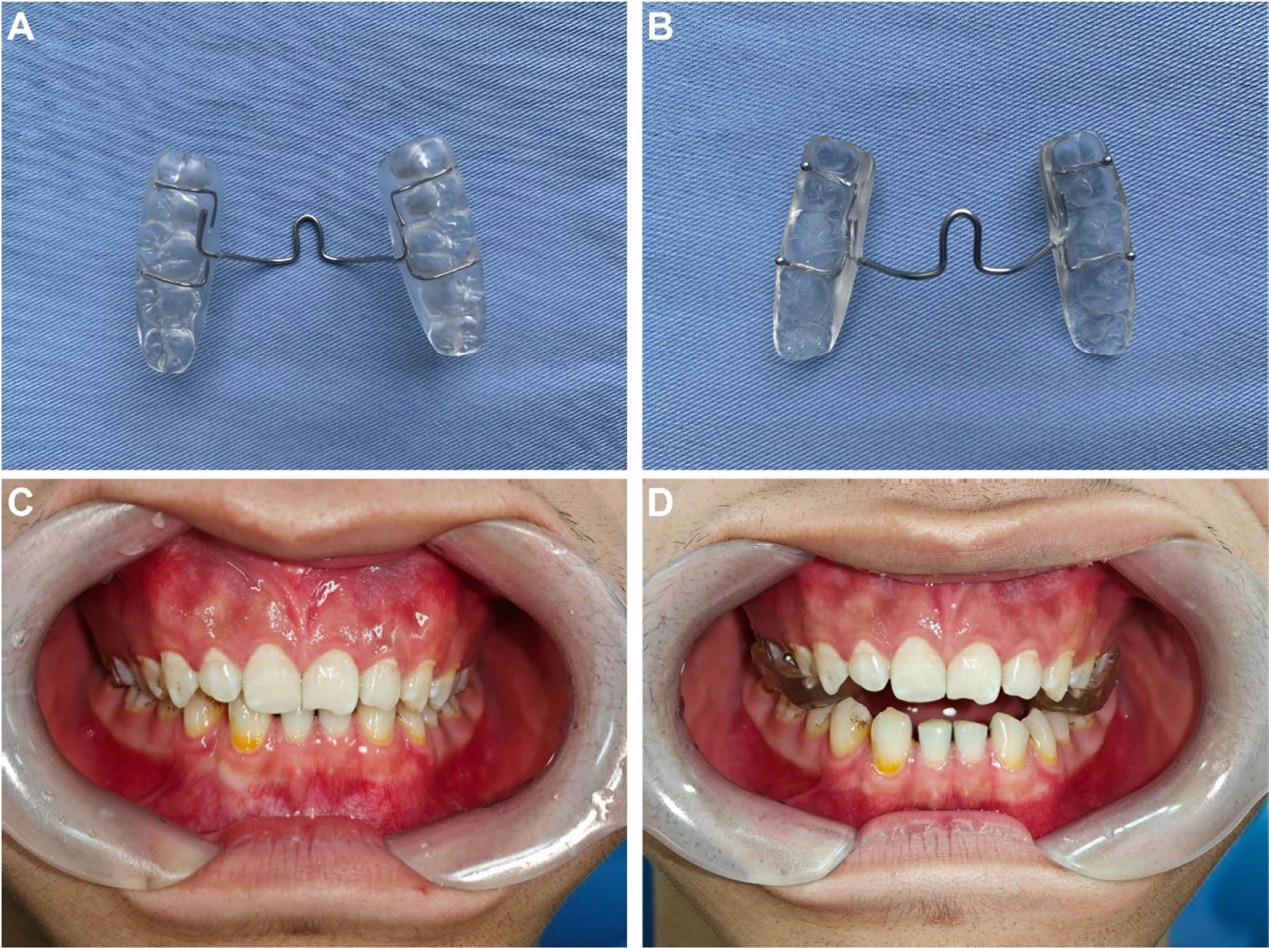
Appearance of the novel posterior occlusal splint and its intraoral application in the temporomandibular disorders (TMD) and deep bite patient. (**A** and **B**) Top and bottom view of the novel posterior occlusal splint. (**C**) Frontal intraoral view of a patient coexisting TMD and deep bite. (**D**) Frontal intraoral view of the same patient wearing the novel posterior occlusal splint

All patients met the following inclusion criteria: (1) presence of TMD symptoms based on the Diagnostic Criteria for Temporomandibular Disorders (DC/TMD) [3]; (2) anterior occlusion diagnosed as deep bite (defined as a vertical overlap between the upper and lower incisors exceeding one-third of the lower incisor crown height) with anterior reverse bite cases excluded (Fig. 1. C); (3) complete permanent dentition from the first premolar to the first molar. Exclusion criteria included: (1) Pulpal pain; (2) Sinusitis; (3) Otitis media; (4) Systemic muscular or joint disorders; (5) Presence of severe congenital, systemic, or hereditary conditions; (6) History of orthognathic surgery, prior TMD treatment, or maxillofacial trauma; (7) Any other conditions deemed unsuitable for inclusion by the investigators.

### Grouping and treatment

All patients were treated with a novel posterior occlusal splint (Fig. 1. A and B). The splint covered the occlusal surfaces of the maxillary posterior teeth, establishing a stable posterior occlusal platform, while anterior overbite was separated due to the thickness of the splint. The posterior teeth on the splint were uniformly elevated by 3 mm, and the splint was clinically adjusted to ensure even contact with the patient’s posterior teeth, without occlusal high spots. After fabrication, the clinician instructed patients on proper procedures for wearing and cleaning the splint (Fig. 1D).

Patients were scheduled for follow-up visits at 1 week, 3 weeks, 1 month, 2 months, 3 months, and 6 months after initial splint wearing. At each visit, the splint was adjusted to maintain even posterior contact and eliminate high spots. Patients were instructed to wear the splint for 10–12 h per day, with wearing time recorded in a daily journal. Clinical examination data were collected at baseline (T0) and at follow-up intervals of 1 month (T1), 3 months (T3), and 6 months (T6) post-treatment.

### Clinical examinations

All clinical examinations were performed by clinicians with over five years of experience in temporomandibular disorder assessment. Prior to data collection, all examiners underwent structured training and calibration based on the DC/TMD [3] to ensure standardized diagnostic procedures. Inter- and intra-examiner reliability was assessed using an independent set of participant data not included in the main analysis. Intraclass correlation coefficients (ICC) were used for continuous variables and Cohen’s kappa (κ) for categorical variables. Both indices showed excellent agreement (ICC/κ ≥ 0.90), indicating high consistency within and between examiners.

All clinical data, including patient history and examination findings, were recorded and securely stored in electronic medical records to ensure data integrity and facilitate quality control. Clinical examination results, including joint sounds, joint pain, intermittent closed lock (ICL), maximum mouth opening (MMO), and the push-back mandibular test, were documented in accordance with the examination methods described in the DC/TMD [3] and our previous study [27].

The clinician placed their fingers on the TMJ and instructed the patient to slowly open their mouth from the intercuspal position to maximum opening, then close it back. (1) while patients performed three repetitions of opening/closing, forward/backward, and lateral mandibular movements. Clicking, popping, or crepitation were recorded as abnormal, as was any joint pain. (2) MMO was recorded as the distance between the upper and lower incisors at full opening. Patients with an MMO < 40 mm were classified as having limited mouth opening, while MMO ≥ 40 mm were classified as without limitation. (3) The push-back mandibular test was conducted by gently pushing the patient’s slightly open jaw upward and backward with the clinician’s palm. Pain in the TMJ area was considered a positive result. (4) Palpation of the TMJ and masticatory muscles was performed at the lateral pole, surrounding areas, temporalis, masseter, and other masticatory muscles, and any positive symptoms were recorded as joint tenderness.

### CBCT imaging measurements of overbite

**Fig. 2:**
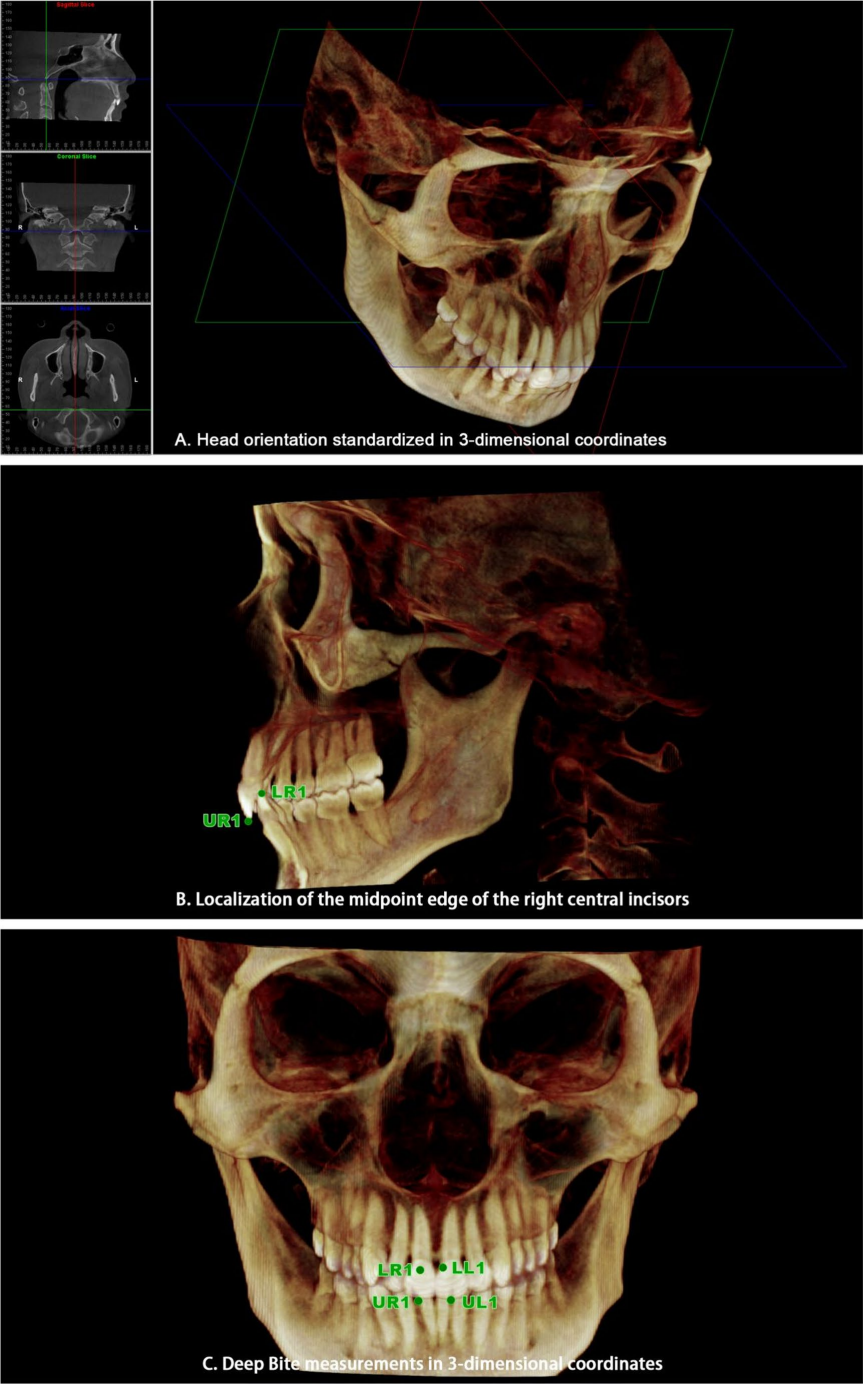
Head orientation and dental landmark identification in reconstructed three-dimensional (3D) cone-beam computed tomography images (CBCT). (**A**) Standardization of head orientation using three reference planes: the horizontal plane, midsagittal plane, and coronal plane. (**B**) Identification of the incisal edge midpoints of the upper right (UR1) and lower right (LR1) central incisors. (**C**) Visualization of all four dental landmarks: UR1, UL1 (upper left), LR1, and LL1 (lower left)

Based on previous studies, Cone-beam computed tomography (CBCT) has been shown to provide reliable and accurate 3D measurements of craniofacial structures and dental landmarks [28]. CBCT scans were performed at T0 and T6 to assist clinicians in evaluating the joint status before and after treatment, guiding therapy planning, and monitoring treatment outcomes. All CBCT data (including raw DICOM files and reconstructed images) were securely stored in the hospital database to ensure data integrity and facilitate quality control. All imaging procedures were conducted at the Department of Radiology, Affiliated Stomatology Hospital of Guangzhou Medical University. Patients were seated in a natural, upright position, with the midsagittal plane perpendicular to the floor and the Frankfurt horizontal plane parallel to the floor. During scanning, patients maintained centric occlusion. CBCT imaging was performed using a NewTom VG Cone-Beam CT system (QR s.r.l., Verona, Italy), with setting of 10.7 mAs and 110 kV. Each scan involved a single 360-degree rotation lasting 3.6 s, producing high-resolution images with a voxel size of 0.25 mm. CBCT data were stored in DICOM format and imported into Dolphin Imaging (version 11.0, Dolphin Imaging & Management Solutions, USA) for three-dimensional(3D) reconstruction and analysis. Standardized head orientation was established by aligning three anatomical reference planes, following previously validated CBCT 3D measurement protocols to ensure reproducibility and accuracy [29]: (1) the horizontal plane (through the right porion and bilateral orbitales); (2) the midsagittal plane (through the nasion and anterior nasal spine, perpendicular to the horizontal plane); and (3) the coronal plane (through the basion, perpendicular to both the horizontal and midsagittal planes). The coordinate system origin was set at the basion. (Fig. 2. A).

Four dental landmarks were identified and defined on all reconstructed 3D CBCT images as the midpoint of the incisal edge of each upper and lower central incisor. These points were designated as UR1 (upper right), LR1 (lower right), UL1 (upper left), and LL1 (lower left) (Fig. 2. B and C). The y-axis coordinates of these landmarks were extracted to assess overbite changes. Each measurement was performed three times by a single investigator, and the average value was used for analysis to minimize intraobserver variability.

To evaluate vertical overbite, the absolute differences in y-axis values between UR1 and LR1 (| ΔY_R_ |), and between UL1 and LL1 (| ΔY_L_ |), were calculated. The average of these two values was defined as the overbite measurement calculated using the following formula:

$$\:Overbite=\frac{\left|\:\varDelta\:{Y}_{R}\:\right|+\left|\:\varDelta\:{Y}_{L}\:\right|}{2}$$

Changes in overbite value between T0 and T6 reflected alterations in vertical overbite.

Both CBCT acquisition and measurement procedures were conducted under blinded conditions: (1) CBCT scans were acquired by radiologic technologists with over five years of clinical experience, who were unaware of the study objectives and patient grouping; (2) 3D measurements were performed by a senior clinician who was also blinded to the study protocol and research hypotheses.

### Statistical analysis

All statistical analyses were conducted using R software. Chi-square tests were employed to compare categorical variable of sex distributions across time points (T1, T3, and T6) relative to baseline (T0). For categorical TMD-related symptoms across time intervals (T0 vs. T1, T1 vs. T3, and T3 vs. T6), chi-square tests were applied when expected cell counts were greater than five; otherwise, Fisher’s exact test was used. Continuous variable of changes in MMO, overbite values and age were analyzed using t-tests. A *p*-value *< 0.05* was considered statistically significant.

## Results

### Characteristics of the patient population and baseline TMD symptoms

**Fig. 3:**
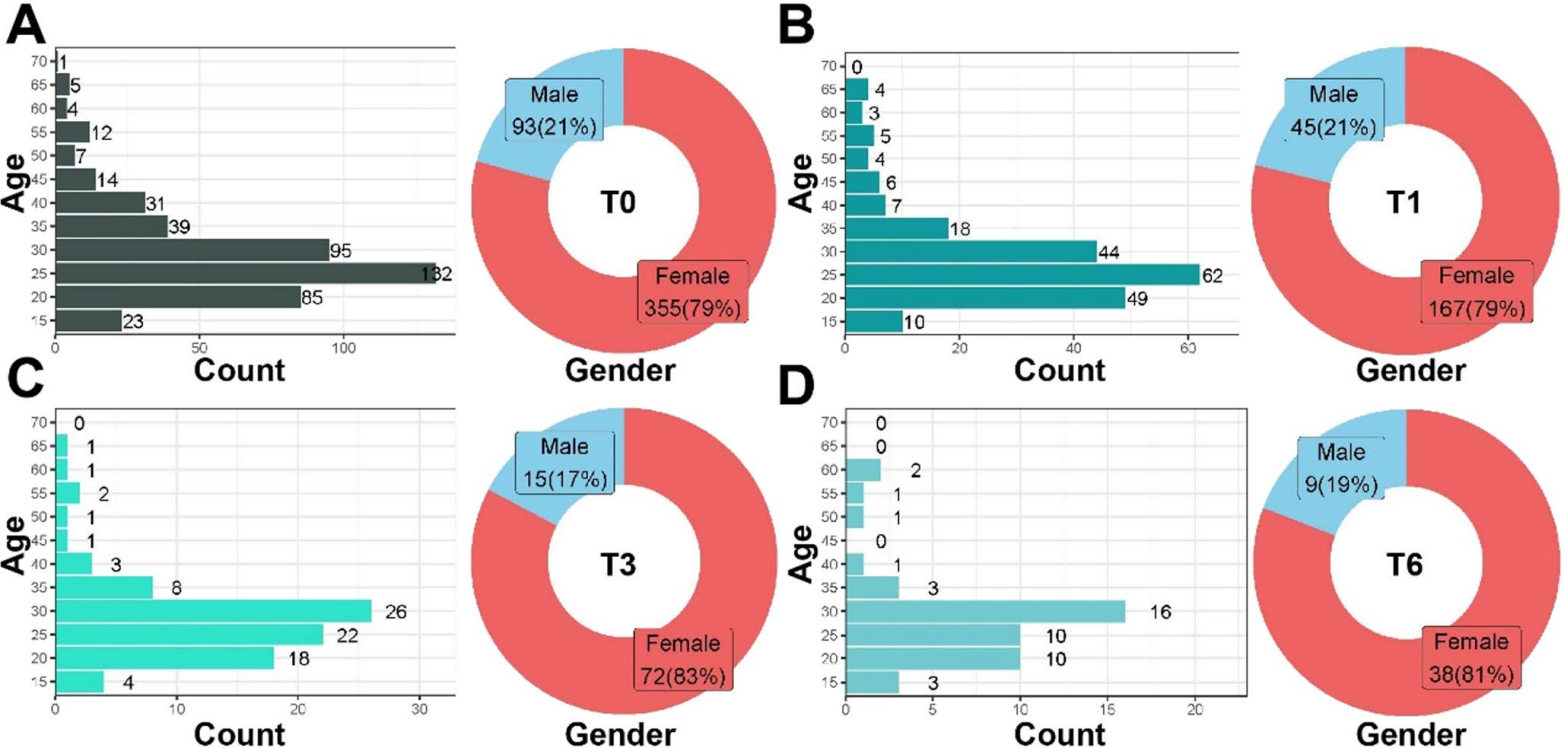
(**A**-**D**) Age distribution, sample size, and gender distribution of patients across time points T0 to T6

A total of 448 patients (355 females and 93 males) with TMD and deep bite were included in this retrospective study. The participants aged 11–66 years (26.53 ± 9.93) (Table 1; Fig. 3. A-D). Patients underwent treatment with the novel posterior occlusal splint, with clinical data recorded at baseline (T0) and follow-ups at 1 month (T1), 3 months (T3), and 6 months (T6). Throughout the study, the 20–35 age group consistently comprised highest proportion of participants, ranging from 69.6% to 76.6% across time points. Chi-square tests comparing age distribution at T1, T3, and T6 with T0 showed *p* > 0.05, indicating no significant differences. Regarding gender distribution, females consistently outnumbered males at all time points, with proportions between 79% and 81%. Chi-square tests comparing gender distribution at T1, T3, and T6 with T0 also yielded *p* > 0.05, indicating no significant differences.

**Table 1:** Age and gender distribution

		T0 (*n* = 448)	T1 (*n* = 212)	T3 (*n* = 87)	T6 (*n* = 47)
Age	<20	23(5.1%)	10(4.7%)	4(4.6%)	3(6.4%)
	20–35	312(69.6%)	155(73.1%)	66(75.9%)	36(76.6%)
	35–40	84(18.8%)	31(14.6%)	12(13.8%)	4(8.5%)
	50–55	23(5.1%)	12(5.7%)	4(4.6%)	4(8.5%)
	>65	6(1.3%)	4(1.9%)	1(1.1%)	0(0%)
	*P*	-	0.710	0.584	0.881
Gender	Male	93(21%)	45(21%)	15(17%)	9(19%)
	Female	355(79%)	167(79%)	72(83%)	38(81%)
	***P***	-	0.972	0.547	0.944

T-test was used to verify the existence of statistical differences between T0 and each timepoint in age

χ^2^ test was used to verify the existence of statistical differences between T0 and each timepoint in gender

Among the 448 patients with TMD combined with deep bite, the pain-related symptoms were analyzed. Of these, 183 patients (41%) reported joint moving pain, 96 patients (21%) experienced joint tenderness, and 26 patients (6%) had a positive result in the push-back mandibular test, indicating TMJ synovitis. Symptoms associated with joint dysfunction included ICL and limited mouth opening, were observed in 35 patients (8%) and 219 patients (49%), respectively. Additionally, 197 patients (44%) experienced joint noise. (Table 2)

**Table 2:** TMD symptoms at different time points

Time		T0(*n* = 448)	T1(*n* = 212)	T3(*n* = 87)	T6(*n* = 47)
Joint Tenderness	**Positive**	96(21%)	25(12%)	7(8%)	4(9%)
	**Negative**	352(79%)	187(88%)	80(92%)	43(91%)
	***P***	-	**0.003** ^#*^	0.455^*##*^	1^*###*^
Joint Moving Pain	**Positive**	183(41%)	61(29%)	14(16%)	6(13%)
	**Negative**	265(59%)	151(71%)	73(84%)	41(87%)
	***P***	-	**0.004** ^#*^	**0.032** ^##*^	0.794^*###*^
Push-back Mandibular Test	**Positive**	26(6%)	3(1%)	1(1%)	0(0%)
	**Negative**	422(94%)	209(99%)	86(99%)	47(100%)
	***P***	-	**0.008** ^@*^	1^@@^	1^*@@@*^
Intermittent Closed Lock (ICL)	**Positive**	35(8%)	14(7%)	4(5%)	2(4%)
	**Negative**	413(92%)	198(93%)	83(95%)	45(96%)
	***P***	-	0.694^*#*^	0.602^@@^	1^@@@^
Maximum Mouth Opening (MMO)	**With limited**	219(49%)	66(31%)	20(23%)	13(28%)
	**Without limited**	229(51%)	146(69%)	67(77%)	34(72%)
	***P***	-	**<0.001** ^#*^	0.203^##^	0.697^###^
Joint Noise	**Positive**	197(44%)	81(38%)	32(37%)	14(30%)
	**Negative**	251(56%)	131(62%)	55(63%)	33(70%)
	***P***	-	0.188^*#*^	0.921^##^	0.533^###^

*#* χ^2^ test was used to verify the existence of statistical differences between T0 and T1

*## χ*^2^ test was used to verify the existence of statistical differences between T1 and T3

*###χ*^2^ test was used to verify the existence of statistical differences between T3 and T6

^@^Fisher exact test was used to verify the existence of statistical differences between T0 and T1

^@@^Fisher exact test was used to verify the existence of statistical differences between T1 and T3

^@@@^Fisher exact test was used to verify the existence of statistical differences between T3 and T6

### TMD symptoms response at different time points after splints treatment

At each time point (T0, T1, T3, and T6), patients were assessed for TMD symptoms. Chi-square tests were used to analyze differences between T0 and T1, T1 and T3, and T3 and T6. When frequencies were less than 5, Fisher’s exact test was applied.

#### Pain-related symptoms at different time points

**Fig. 4:**
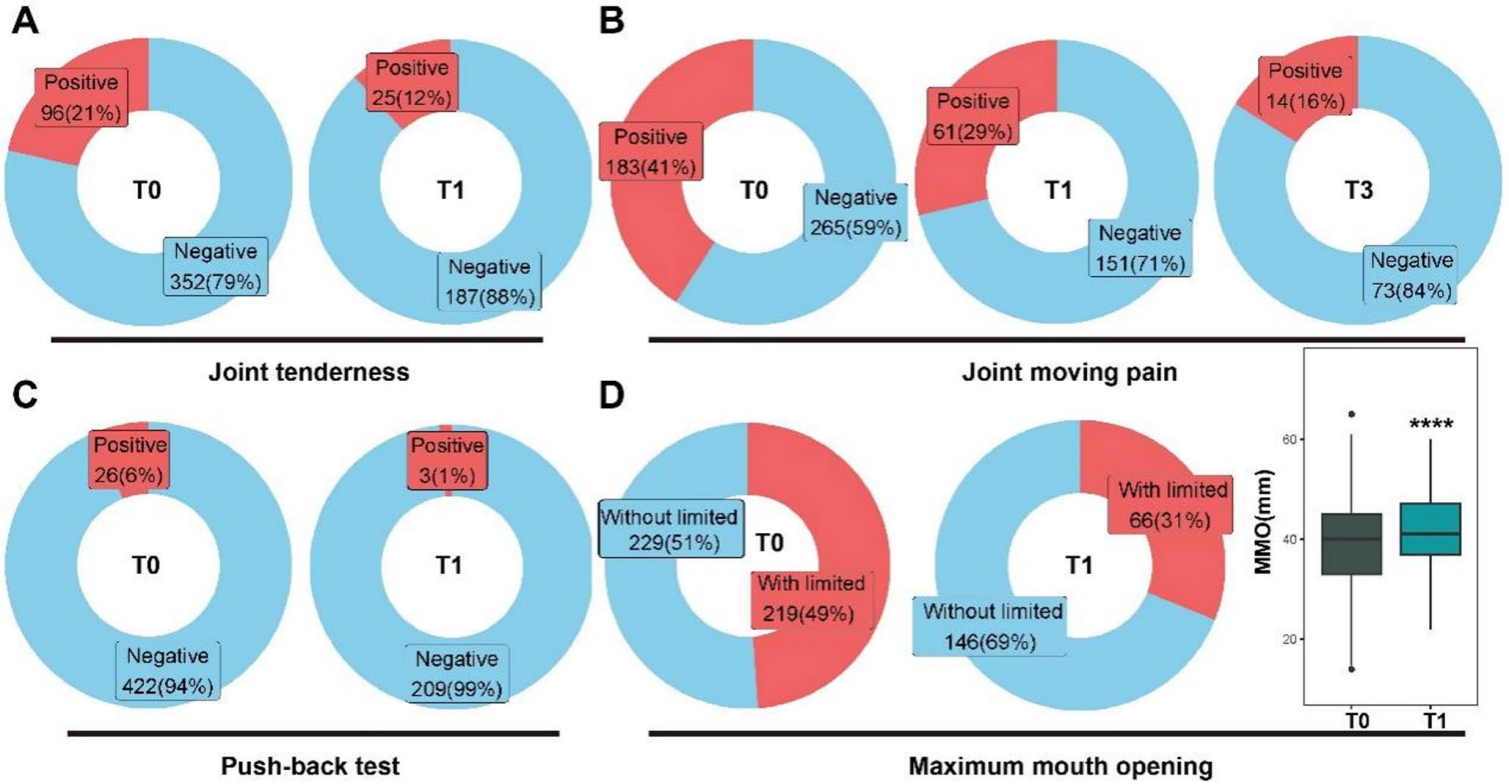
Time-course analysis of symptom improvement and proportion of patients with positive clinical signs. (**A**) Proportion of patients with relief from joint tenderness across treatment time points. (**B**) Improvement in joint movement-related pain during treatment. (**C**) Reduction in positive push-back mandibular test results over time. (**D**) Changes in maximum mouth opening (MMO) indicating functional improvement. ****p < 0.0001 indicates statistically significant differences compared to baseline in paired t-test

For patients with TMD and deep bite, wearing a posterior occlusal splint resulted in gradual improvements in pain-related symptoms over time (Table 2). At baseline (T0), 21% of patients had joint tenderness, which decreased to 12% at T1 and 9% at T6, respectively. The reduction from T0 to T1 was statistically significant (*p* = 0.003) (Table 2; Fig. 4. A). However, no significant changes were observed between T1 and T3 (*p* = 0.455) or T3 and T6 (*p* = 1). Joint moving pain, which affected 41% of patients at T0, decreased to 13% at T6. Significant reductions were observed from T0 to T1 (*p* = *0.004*) and T1 to T3 (*p* = *0.032*) (Table 2; Fig. 4. B). No significant change was noted between T3 and T6 (*p* = *0.794*). The push-back mandibular test showed a decrease in positive cases from 6% at T0 to 0% at T6, indicating improved TMJ function. The improvement from T0 to T1 was significant (*p* = *0.018*) (Table 2; Fig. 4. C), while subsequent changes were not statistically significant.

#### TMJ dysfunction symptoms at different time points

In addition to pain-related symptoms, joint dysfunction issues in patients with TMD and deep bite should also be considered. These symptoms provide valuable insights into treatment effectiveness and the overall condition of the TMJ. Initially, ICL was observed in 8% of patients, which decreased to 4% by T6 (Table 2). Across all time point comparisons from T0 to T6, the *p*-values were all greater than 0.05, indicating no significant differences in ICL changes among patients. At T0, 49% of patients had limited mouth opening. This improved to 31% at T1 and 28% at T6 (Table 2). A chi-square test showed a significant improvement from T0 to T1 (*p* < 0.001), with the MMO increasing (Table 2; Fig. 4. D). However, no significant differences were observed between T3 and T1 or between T6 and T3 (*p* = 0.203,*p* = 0.697).

#### Other TMD-related symptoms at different time points

Joint noises, initially present in 44% of patients, decreased to 30% at T6, indicating improved mandibular stability (Table 2). Although there was a reduction in joint noises from T0 to T6, no statistically significant differences were observed at any time point comparisons.

### Prognosis in TMD symptoms during splints treatment

To access the prognosis of TMD symptoms over time, we tracked the patients who continuously wore the splint from stage T0 to stage T6. we tracked patients who consistently wore the splint from stage T0 to T6. Symptom progression was visualized using pie charts and Sankey diagrams to illustrate the transitions between positive and negative symptom states across the different time points. Patients transitioning from positive to negative symptoms were categorized as experiencing symptom relief, while those shifting from negative to positive were considered to have symptom recurrence.

#### Prognosis in pain-related symptoms during treatment

**Fig. 5:**
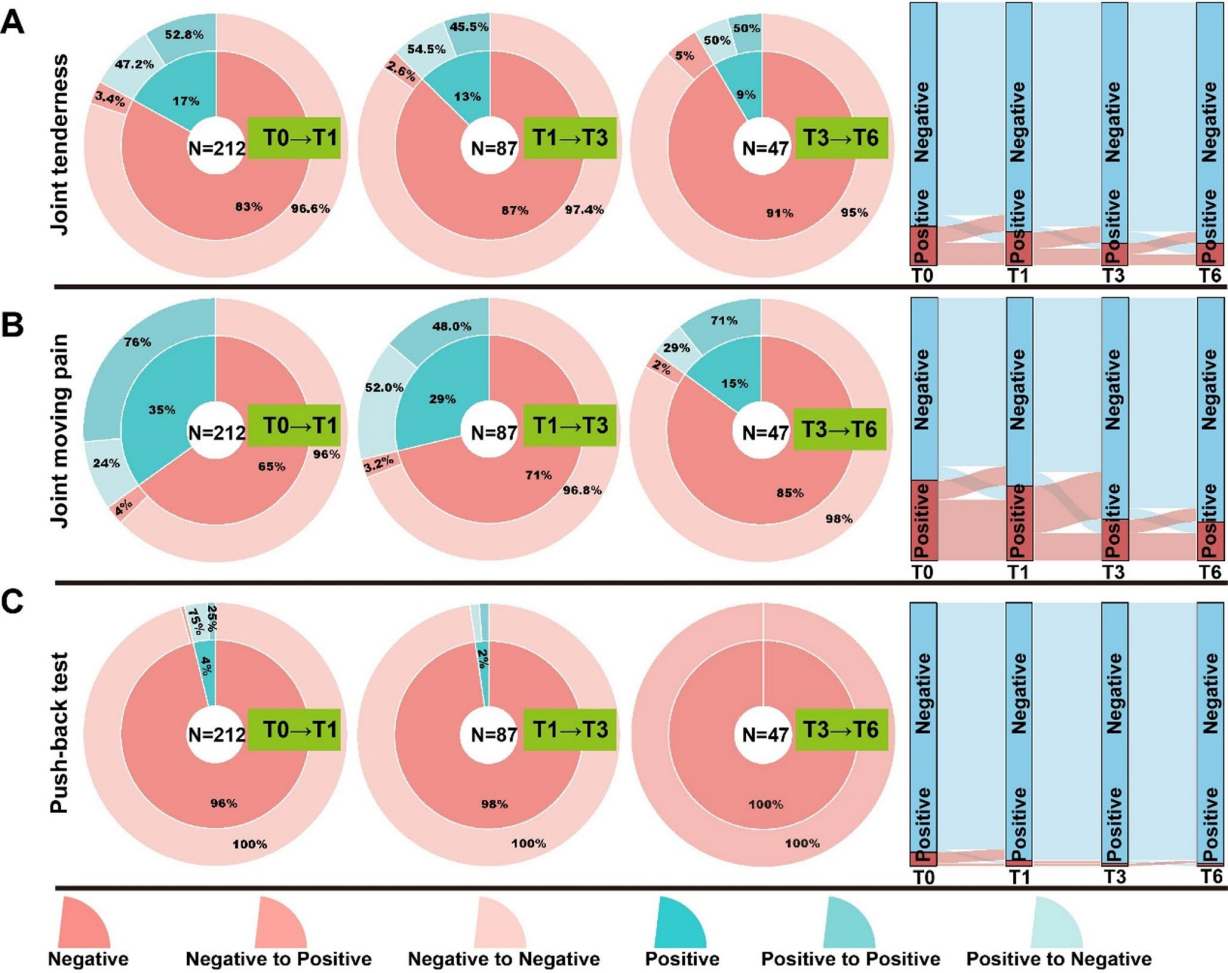
Prognosis in pain-related symptoms during splint treatment. (**A**) Changes in the proportion of patients experiencing joint tenderness over time. (**B**) Variation in joint moving pain relief across different treatment stages. (**C**) Decrease in positive push-back mandibular test results, reflecting alleviation of joint pain

Among patients who completed splint therapy from T0 to T6, the proportion reporting relief from joint tenderness remained relatively consistent, ranging from 47.2% to 54.5%. The recurrence rate for this symptom remained low, between 2.6% and 5% (Fig. 5. A). For joint movement-related pain, symptom relief was reported in 24% of patients at T1, increasing to 29% by T6, with the peak relief rate occurring at T3 (52%). Symptom recurrence ranged from 2% to 4% (Fig. 5. B). Notably, among patients who initially tested positive in the push-back mandibular test, 75% showed relief by T1, and all tested negative by the six-month follow-up, indicating complete resolution of this symptom (Fig. 5. C).

#### Prognosis in joint dysfunction symptoms during treatment

**Fig. 6:**
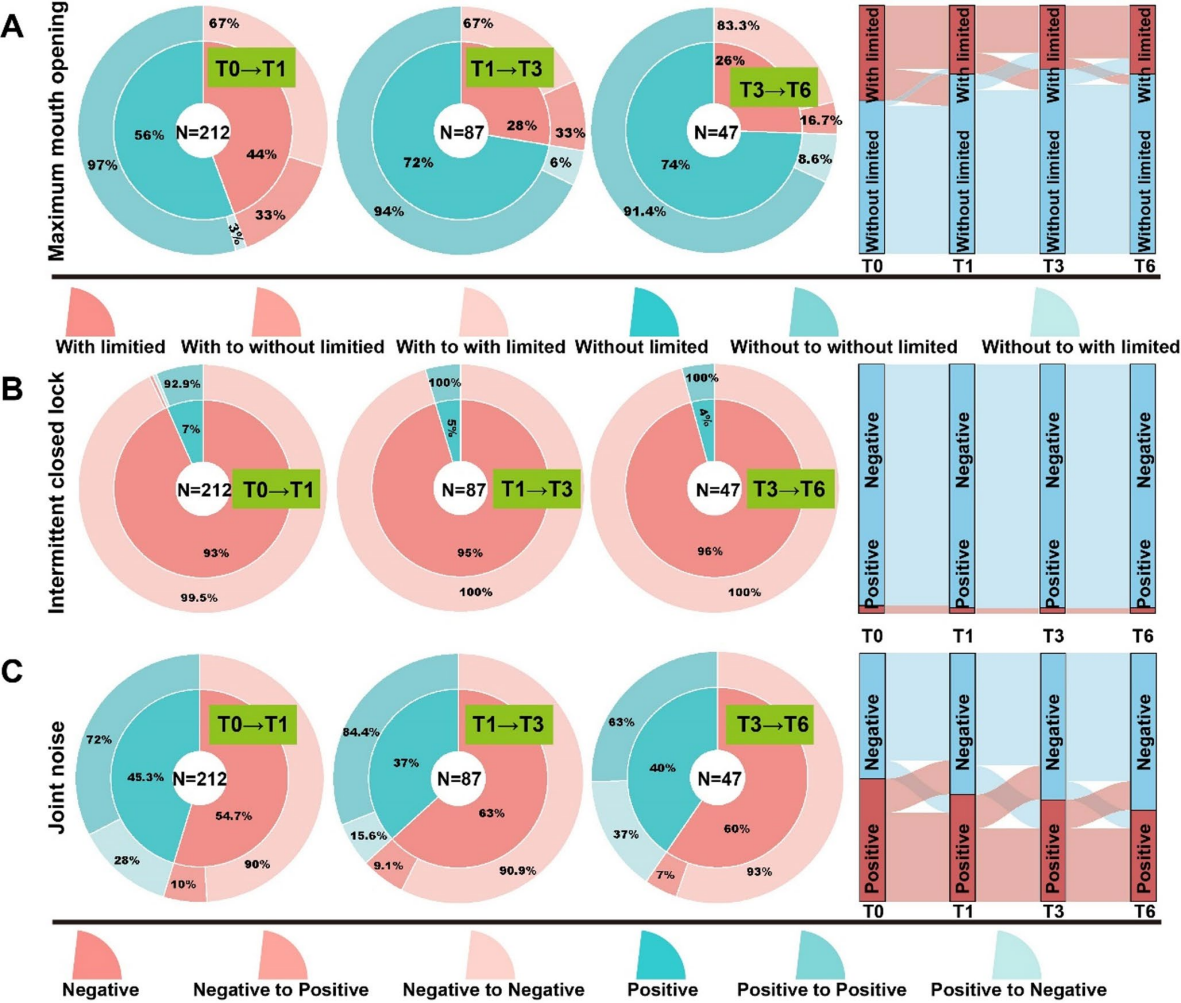
Prognosis in joint dysfunction and joint noises symptoms during splint treatment. (**A**) Improvement and recurrence trends in maximum mouth opening (MMO) across treatment stages. (**B**) Changes in the proportion of patients with intermittent closed lock (ICL) throughout the treatment timeline. (**C**) Trends in improvement and recurrence of joint noises from stage T0 to T6

For patients completing treatment through stage T6, 33% showed an improvement in MMO at both T1 and T3. However, this improvement declined to 16.7% by T6. Meanwhile, the recurrence rate of restricted MMO increased over time, rising from 3% at T1 to 6% at T3 and reaching 8.6% at T6 (Fig. 6. A). In cases with initial ICL, 7.1% of patients showed improvement at T1, with no further improvement observed at later time points (T3 or T6). Only a minimal recurrence rate (0.5%) was observed at T1 (Fig. 6. B).

#### Prognosis in joint noises during treatment

Improvements in joint noises followed a non-linear trend. From T0 to T6, the proportion of patients showing improvement initially decreased from 28% to 15.6%, before increasing again to 37%. Symptom recurrence related to joint noises ranged from 7% to 10% during the treatment period (Fig. 6. C).

### Overbite changes

**Fig. 7:**
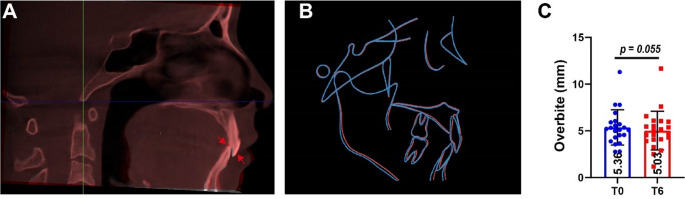
Overbite changes based on cone-beam computed tomography (CBCT) superimposition and measurements. Representative images. (**A**) Superimposed CBCT images at T0 (red) and T6 (white) after head orientation calibration in Dolphin software. (**B**) Lateral cephalograms reconstructed from CBCT at T0 and T6 with anatomical outlines. The red outline corresponds to T0, and the blue outline to T6, high lighting overbite changes of the central incisors. (**C**) Quantitative changes in overbite measurement (paired t-test)

Three-dimensional overbite measurements based on CBCT data were conducted were conducted for 22 patients at T0 and T6. Changes in central incisor overbite were assessed on the sagittal plane, with a slight decrease observed. (Fig. 7. A and B). The mean overbite decreased from 5.36 ± 1.89 mm at T0 to 5.03 ± 2.07 mm at T6; however, this change was not statistically significant (*p* = 0.055) (Fig. 7. C).

## Discussions

The key findings of this study are that the novel posterior occlusal splint rapidly relieves pain-related TMD symptoms, improves limited mouth opening, and that multi-time-point follow-up with 3D CBCT measurements reveals symptom fluctuation patterns and occlusal changes. This not only provides valuable guidance for prognosis assessment and patient selection but also supports the splint’s role as an effective conservative treatment option for patients with TMD and deep bite.

In this study, we retrospectively analyzed clinical records of patients with TMD and deep bite who were treated with a novel posterior occlusal splint. Compared with traditional splints, this newly designed appliance features reduced volume, improved comfort and patient compliance, and enhanced therapeutic efficacy [30]. Our findings demonstrated significant relief of TMD symptoms and improved overall prognosis in this patient population. Among them, patients presenting TMD and deep bite showed significant improvement in pain or limited mouth opening discontinued splint use. For those who did not experience notable symptom relief after six months, splint therapy was stopped and alternative treatments were pursued. The marked decrease in patient numbers at time point T6 was mainly due to the resolution of primary symptoms through short-term splint use.

Deep bite is a type of malocclusion characterized by vertical discrepancy, and is widely recognized as a contributing factor in TMD development [18, 32–34]. Reduction in the vertical distance between the anterior teeth may cause disturbances in the TMJ through condylar overclosure, altering the loading pattern of the joint. This may lead to increased intra-articular pressure and stress concentration, which can trigger disc displacement and structural damage, gradually impairing joint function [4]. The risk is even higher when accompanied by lingual inclination of the maxillary incisors [17].

In present of lingually inclined maxillary incisors, protrusive and lateral mandibular movements require an exaggerated rotational range to overcome anterior occlusal interferences. This leads to a lack of coordination between the direction of mandibular movement and the contraction vectors of the elevator muscles of mastication. The resulting biomechanical discrepancy may lead to overstretching and laxity of the posterior ligament of the TMJ articular disc, contributing to masticatory muscle dysfunction [35, 36]. During mandibular closure, the weakened tension within the posterior ligament hinders its capacity to reposition the disc posteriorly, subsequently compromising disc stability and exacerbating TMJ dysfunction [37]. Clinically, these changes may present as joint pain during mandibular movements, restricted mouth opening, and mandibular deviation or asymmetry.

The persistent presence of a deep bite can lead to excessive wear of posterior cusps, further reducing the vertical dimension of occlusion. This progressive deterioration increases the mechanical load on the masseter and posterior temporalis muscles, imposing abnormal stress on the articular disc [17]. Such stress may trigger muscle dysfunctions, including spasm of the lateral pterygoid muscle, which in turn disrupts the disc-condyle relationship. Clinically, these alterations may manifest as typical signs and symptoms of TMD, such as joint noises, masticatory pain, and masticatory discomfort [18].

Consistent with these mechanisms, our patient data revealed that individuals with TMD and deep bite commonly presented with pain-related symptoms: 41% experienced pain during mandibular movement, 21% had joint tenderness, and 6% reported pain during the mandibular push-back test. Additionally, 49% presented with limited mouth opening, and 44% exhibited joint noises. These symptoms are often attributable to disc-condyle discrepancies, which, if untreated, may lead to progressive condylar resorption and joint degeneration [38–40]. Thus, effective management of cases with both TMD and deep bite requires restoration of occlusal vertical dimension, elimination of anterior occlusal interferences, and interruption of the pathological feedback loop perpetuated by malocclusion.

Our results showed that the novel posterior occlusal splint effectively was particularly effective in alleviating pain-related TMD symptoms within the first three months of treatment. Although previously reported stabilization, soft, flat, and pivot splints also demonstrated effectiveness in reducing pain intensity and painful episodes in adult TMD patients, especially those without severe osteoarthritis, these studies did not specifically show that significant pain relief could occur within the first three months [41, 42]. Approximately 40–50% of patients experienced improved joint tenderness across different time points. Among those with joint pain during mandibular movement, the highest symptom relief rate (52%) was recorded at the mid-treatment stage (T3). Additionally, the recurrence rate remained low (< 5%), suggesting sustainable therapeutic effects. These outcomes may be attributed to increased joint space, reduced occlusal load, and alleviation of masticatory muscle tension and spasms.

This splint also significantly improved MMO, with a notable progress observed within the first month. By six months, the proportion of patients with restricted mouth opening had declined from 44% to 26%. However, an 8.6% recurrence rate was noted with prolonged wear, indicating that the splint may be most effective as a short-term intervention. It appears particularly beneficial in reducing anterior occlusal interferences and improving mandibular mobility. In patients with anterior disc displacement without reduction (ADDwoR), however, the splint was less effective in restoring disc-condyle relationships, suggesting the need for adjunctive treatment strategies.

In cases of ICL, this splint exhibited a stabilizing effect. Although the overall improvement rate was modest (7.1% at T1), the recurrence rate remained minimal. Given the limited sample size, further studies are required to assess long-term efficacy. In contrast, joint noises exhibited variable trends with no consistent improvement throughout the treatment period. Since joint noises alone may not accurately reflect clinical progression-and may, in some cases, indicate worsening disc displacement-they should not be adopted as a sole metric for clinical evaluation. Comprehensive assessment incorporating clinical symptoms, functional evaluation, and imaging is essential for accurate diagnosis and appropriate treatment planning.

In present study, after six months treatment with the novel posterior occlusal splint, the mean overbite value showed a slight decreased, and the change was not statistically significant. This outcome may be attributed to an insufficient sample size and substantial variability between group. Nevertheless, an improvement in deep bite was observed in 64% of patients (14 out of 22) following this splint treatment. This improvement may be related to changes in mandibular position induced by splint therapy. Studies have shown that treatment with full-arch stabilization splints is often associated with a reduction in overbite and a tendency toward anterior open bite [43]. This reduction typically reflects a change in mandibular position-usually a clockwise rotation-rather than true opening of the bite [44–46]. The novel posterior occlusal splint, as a modified design of the traditional stabilization splint, may share a similar mechanism, thereby contributing to the reduction in overbite.

Occlusal changes resulting from alterations in mandibular position are often reversible [47]. However, prolonged use of occlusal splints that contact only part of the opposing dentition may result in permanent occlusal alterations [48]. To avoid undesirable tooth intrusion or extrusion, long-term use (> 12 months) should be undertaken with caution and closely monitored [49]. Moreover, previous studies have shown that treatments such as stabilization splints can induce adaptive remodeling of the condyle after 6–12 months of use, including positional changes and alterations in joint space, indicating that prolonged splint therapy may also trigger structural adaptations [50]. Findings from this study suggest that use within six months is relatively safe. Nonetheless, the potential risk of irreversible occlusal changes and condylar remodeling associated with extended wear of this novel posterior occlusal splint requires further investigation.

Notably, our study provides new insights in two key aspects. First, while prior research on occlusal splints mainly focused on symptom relief, few studies tracked symptom fluctuation and prognosis during the splint-wearing process. By monitoring patients at multiple time points, we observed patterns of symptom recurrence and resolution, providing clinically relevant guidance on disease progression and timing of interventions. Second, previous CT-based studies in TMD primarily measured joint space changes, rarely quantifying occlusal modifications. Using precise 3D CBCT measurements, we evaluated overbite changes after splint therapy, offering objective evidence of occlusal alterations and enhancing understanding of how splints affect both joint function and occlusion.

In conclusion, the novel posterior occlusal splint significantly alleviated joint symptoms (including pain-related symptoms and limited mouth opening) in patients with TMD and deep bite. However, its effectiveness may vary depending on individual anatomical and pathological factors. For patients with suboptimal responses, additional interventions such as intramuscular injections, intra-articular therapies, orthodontic treatment, or surgery may be required. Occlusal splints that contact only specific opposing teeth may provide short-term relief from acute TMJ pain. However, prolonged use can lead to unwanted tooth movement, such as extrusion of non-contacting teeth and intrusion of contacting teeth.

Despite these promising results, several limitations should be acknowledged. This study was retrospective and conducted at a single center, which may introduce selection bias and dependence on existing medical records, some of which lacked detailed clinical information such as prior treatment history, limiting generalizability. No control group was included, preventing direct comparison with conventional stabilization splints or other conservative approaches. The six-month follow-up period restricted evaluation of long-term efficacy, safety, and potential cumulative effects. In addition, the relatively small sample size limited the ability to perform subgroup analyses by age or TMD subtype.

Future research should address these limitations by employing multicenter, prospective, controlled designs with larger sample sizes and conventional splint comparators. Extended follow-up beyond one year is warranted to evaluate long-term outcomes. Stratified analyses by age and TMD subtype may help refine patient selection criteria. Advanced imaging techniques could further elucidate the effects of splint therapy on joint anatomy and occlusion, ultimately informing optimized treatment protocols and improving clinical outcomes for patients with TMD and deep bite.

## Conclusion

1. The novel posterior occlusal splint demonstrates promising therapeutic efficacy in patients with both TMD accompanied by deep bite.
2. It effectively provided short- to medium-term pain relief and improvements in mandibular function.
3. However, its effects on joint noises and deep bite are limited, and the long-term therapeutic benefits remain uncertain given this study’s follow-up duration.

## Data Availability

The corresponding author ***D.DS. Dr. W. Cao*** had full access to the data in the study and takes responsibility for the integrity of the data and the accuracy of the data analysis. The data and material that support the findings of this study are available from the corresponding author upon reasonable request.

## Abbreviations

ADDwoR: Anterior Disc Displacement without Reduction
CBCT: Cone-Beam Computed Tomography
DC/TMD: Diagnostic Criteria for Temporomandibular Disorders
ICL: Intermittent Closed Lock
MMO: Maximum Mouth Opening
TMD: Temporomandibular Disorders
TMJ: Temporomandibular Joint
ICC: Intraclass Correlation Coefficients
χ² test: Chi-square test
